# Plasma exosomes from children with juvenile dermatomyositis are taken up by human aortic endothelial cells and are associated with altered gene expression in those cells

**DOI:** 10.1186/s12969-019-0347-0

**Published:** 2019-07-12

**Authors:** Kaiyu Jiang, Rie Karasawa, Zihua Hu, Yanmin Chen, Lucy Holmes, Kathleen M. O’Neil, James N. Jarvis

**Affiliations:** 1 Department of Pediatrics, University at Buffalo Jacobs School of Medicine and Biomedical Sciences, Buffalo, NY USA; 2 Department of Frontier Medicine, St. Marianna University School of Medicine, Kawasaki, Japan; 3 Center for Computational Research, University at Buffalo Jacobs School of Medicine and Biomedical Sciences, Buffalo, NY USA; 4 Department of Pediatrics, Indiana University School of Medicine, Indianapolis, IN USA; 5 Genetics, Genomics, & Bioinformatics Program, University at Buffalo Jacobs School of Medicine and Biomedical Sciences, Buffalo, NY USA

**Keywords:** Juvenile dermatomyositis, Plasma exosomes, Endothelial cells, Gene expression, RNA-seq, miRNA-seq

## Abstract

**Background:**

The pathology of juvenile dermatomyositis (JDM) is characterized by prominent vessel wall and perivascular inflammation. This feature of the disease has remained unexplained and under-investigated. We have hypothesized that plasma exosomes, which play an important role in inter-cellular communication, may play a role in the vascular injury associated with JDM.

**Objective:**

To characterize the circulating exosomes of children with JDM and determine whether the small RNA cargoes within those exosomes are capable of altering transcriptional programs within endothelial cells.

**Design/methods:**

We purified exosomes from plasma samples of children with active, untreated JDM (*n* = 6) and healthy controls (*n* = 9). We characterized the small RNA cargoes in JDM and control exosomes by RNA sequencing using the Illumina HiSeq 2500 platform. We then incubated isolated exosomes from healthy controls and children with JDM with cultured human aortic endothelial cells (HAEC) for 24 h. Fluorescence microscopy was used to confirm that both control and JDM exosomes were taken up by HAEC. RNA was then purified from HAEC that had been incubated with either control or JDM exosomes and sequenced on the Illumina platform. Differential expression of mRNAs from HAEC incubated with control or JDM exosomes was ascertained using standard computational methods. Finally, we assessed the degree to which differential gene expression in HAEC could be attributed to the different small RNA cargoes in JDM vs control exosomes using conventional and novel analytic methods.

**Results:**

We identified 10 small RNA molecules that showed differential abundance when we compared JDM and healthy control exosomes. Fluorescence microscopy of labeled exosomes confirmed that both JDM and control exosomes were taken up by HAEC. Differential gene expression analysis revealed 59 genes that showed differential expression between HAEC incubated with JDM exosomes vs HAEC incubated with exosomes from controls. Statistical analysis of gene expression data demonstrated that multiple miRNAs exerted transcriptional control on multiple genes with HAEC.

**Conclusions:**

Plasma exosomes from children with active, untreated JDM are taken up by HAEC and are associated with alterations in gene expression in those cells. These findings provide new insight into potential mechanisms leading to the targeting of vascular tissue by the immune system in JDM.

**Electronic supplementary material:**

The online version of this article (10.1186/s12969-019-0347-0) contains supplementary material, which is available to authorized users.

## Background

Prominent vascular changes and perivascular inflammation are distinct clinical [1] and pathological [2, 3] features of juvenile dermatomyosits (JDM) and underlie much of the morbidity associated with the disease [3]. The mechanisms through which the blood vessels are targeted by the immune system in this disease remain incompletely understood. A prominent type 1 interferon-induced gene expression signature is seen in both affected muscle tissue and peripheral blood of children with JDM [4], and there is accumulating evidence that type 1 interferons can directly harm the vasculature [5], but the specific relationship between vascular damage and the interferon signature in dermatomyositis remains uncertain [6]. We have recently reported that endothelial cells in JDM are targeted by a brisk autoantibody response [7], but we were unable to ascertain whether such autoantibodies represent a primary driver of the pathology or a response to immunologically-injured endothelium. Thus, the question of how the vasculature becomes targeted or injured in JDM remains unanswered.

Exosomes are one of several families of microparticles that are released from cells either after specific stimuli or during cellular apoptosis [8]. We are now coming to understand that these subcellular components contain small, non-coding RNA molecules that form a previously unrecognized level of transcriptional control in mammals and possibly in simpler organisms [9]. The RNA molecules contained within exosomes and other microparticles are, among other things, a mechanism through which cells of the immune system communicate with one-another [10]. These microparticles are abundant in serum and plasma and have been a source of considerable interest as biomarkers in a broad range of diseases [11–13]. Since the endothelium is in intimate contact with plasma, and therefore circulating microparticles such as exosomes, we believe that understanding more about the composition and biological behavior of circulating exosomes may yield new insights into basic disease mechanisms in JDM.

In this paper, we describe the small RNA contents of exosomes purified from the plasma of children with JDM and describe their interactions with endothelial cells in an in vitro model. We believe these studies provide new insights into the complex mechanisms that appear to underlie JDM and very likely other forms of vasculitis.

## Methods

### Patients and patient specimens

Plasma was obtained from 6 children with newly diagnosed, untreated JDM who presented at the University of Oklahoma Pediatric Rheumatology Clinic between 2007 and 2011. All children had weakness, rash, elevated serum concentrations of CPK, AST, and ALT and altered nailfold capillaries. There were 4 girls (age range 3–10 years) and 2 boys (ages 14 and 15 years) whose plasma samples were used for these studies. Plasma was also obtained from healthy children (HC; *n* = 9) who were patients at Hodge Pediatrics, a general pediatrics clinic operated by the Women and Children’s Hospital of Buffalo Hodge Pediatrics Clinic. Among the HC, there were 3 boys (ages 8–13 years) and 6 girls (ages 6–14 years). HC were excluded if they were taking antibiotics or systemic steroids, had an underlying autoimmune condition such as type 1 diabetes or autoimmune thyroiditis, had asthma and on a leukotriene receptor antagonist or a medium or high dose inhaled corticosteroid, had invasive surgery in the previous 3 weeks, were obese with a BMI at greater than the 95th percentile for their age, or had temperature greater than 38.0 within the previous 36 h.

All research procedures were reviewed and approved by the Institutional Review Boards (IRB) of the University of Oklahoma Health Sciences Center and the University at Buffalo Jacobs School of Medicine & Biomedical Sciences. All research was performed in compliance with the IRB-approved protocol. Written informed consent was obtained from the parents or guardians of all subjects, and, where appropriate, assent was obtained from children in accordance with IRB guidelines at each institution.

Blood was drawn into CPT tubes (Becton Dickinson, Cat # 362761, Franklin Lakes, NJ) and brought to the laboratory for processing within an hour of being obtained. Plasma was separated from cells by centrifugation and stored at -80 °C until used.

### Purification of exosomes from plasma

Exosomes were isolated from 500 μL of plasma using the ExoQuick exosome precipitation solution (System Biosciences, Mountain View, CA, USA) according to the manufacturer’s methods. Briefly, the plasma was incubated with thrombin liquid suspension (500 U/mL) (System Biosciences) for 5 min while mixing at room temperature. After centrifugation at 10,000 rpm for 5 min, the supernatant was then filtered through a 0.2 μm Spin X column filtration system (Sigma) to remove any cell debris and particles larger than 200 nm. The filtrate was mixed with 120 μL of ExoQuick solution and RNase A (Sigma, St. Louis, MO, USA) to a final concentration of 10 μg/mL. The mixture was kept at 4 °C overnight and then further mixed with 100 units/mL of murine RNase inhibitor (NEB) before centrifugation at 13,000 rpm for 2 min. The pellet was washed in phosphate-buffered saline (PBS), then the PBS was removed.

### Exosome identification

To verify that the material prepared as described above represented plasma exosomes, Western blot assay was performed for a common exosome marker, CD63. Protein was loaded on 10% SDS-PAGE gels. After electrophoretic separation, proteins were transferred to a nitrocellulose membrane (Bio-Rad). The membranes were blocked with 5% milk in 1× TBS with 0.05% Tween 20 (TBST) for 1 h at room temperature. Primary antibodies (rabbit anti-human CD63 antibody, System Biosciences, Mountain View, CA, USA) were added to the membrane and incubated overnight at 4 °C with gentle agitation. The membranes were further washed with TBST and incubated for 1 h with horseradish peroxidase (HRP)-conjugated goat anti-rabbit IgG (System Biosciences). The immunoreactive proteins were detected using an enhanced chemiluminescence substrate kit (SuperSignal, Pierce, Rockford, IL) according to the manufacturer’s instructions.

Further verification that our isolation procedures had resulted in purification of plasma exosomes was undertaken using transmission electron microscopy. Exosome enriched fractions from thrombin treated plasma were fixed using 2% glutaraldehyde at 4 °C overnight. The following day, the fixed exosomes were deposited on the surface of a 400-mesh carbon-coated grids, air-dried, stained with uranyl acetate, and transmission election microscope (TEM) images were recorded with a JEM-100CX II transmission electron microscope (JEOL, Peabody, MA, USA).

### Purification of RNA from isolated exosomes

Exosomal RNA was extracted using a miRNeasy Micro Kit (QIAGEN, Valencia, CA, USA). Exosome pellets was mixed with 700 μL QIAzol lysis buffer, and the mixture was processed according to the manufacturer’s standard protocol. To eliminate potential co-precipitated DNA, the column-bound RNA was treated with DNase I for 15 min at room temperature. The RNA was then eluted with 14 μl of DNase- and RNase-free water. RNA quality and quantity were estimated by Agilent Bioanalyzer 2100 using a Small RNA Chip (Agilent, Santa Clara, CA).

### Uptake of exosomes by cultured human aortic endothelial cells (HAEC) and purification of total RNA from HAEC

HAECs were cultured in 4-well Lab-Tek chamber coated with collagen in Medium 200 plus Low Serum Supplement (Life Technologies) for 24 h. Exosomes from plasma were labeled with PKH67 (Sigma) for 30 min followed by PBS washes. HAECs were incubated with PKH67 labeled exosomes for 24 h. HAECs were fixed by 4% formaldehyde and the slide was mounted using ProLong Gold antifade reagent with DAPI (Life Technologies). The chamber slides and exosomes were visualized with a fluorescent imaging microscope (Zeiss).

In order to determiner the biological effects of JDM exosomes after their uptake by HAEC, we cultured HAECs in 6-well plates coated with collagen in Medium 200 plus Low Serum Supplement. When HAEC cells had approximate 85% confluence, they were incubated with exosomes from JDM (*n* = 4) or healthy control plasma (*n* = 4) for 24 h. HAEC total RNA was isolated using Trizol and RNeasy Mini Kit (Qiagen) as described previously [14].

### RNA sequencing

Small RNA libraries were constructed following the manufacturer’s instructions using the TruSeq Small RNA Library Prep Set from Illumina. RNA libraries were amplified by 11 PCR cycles using Illumina compatible index primers. Size selection of the amplified libraries was done using Blue Pippin 3% gel cassettes (Sage Sciences). cDNA fragments from 140 to 160 bp (the length of miRNA inserts plus the 3′ and 5′ adaptors) were eluted. cDNA libraries were quantified using Picogreen Assay (Invitrogen) and Library Quantification kit (Kapa Biosystems, Wilmington, MA). Agilent Bioanalyzer 2100 Hi Sensitivity DNA chip was used to confirm the sizes of the cDNA libraries. The cDNA products were then sequenced using the Illumina HiSeq2500 at the UB Genomics and Bioinformatics Core Facility (Buffalo, NY). Ten sequencing libraries were pooled into a single sequencing lane.

mRNA libraries were constructed at the UB Genomics and Bioinformatics Core Facility (Buffalo, NY) using the TruSeq RNA Library Preparation Kit. All samples were subjected to 50-cycle, single-read sequencing in the HiSeq 2500 (Illumina).

### Analysis of small RNA cargoes in exosomes from RNAseq data

For miRNA sequence analysis, read counts for individual miRNAs were obtained for each sample using miRDeep* [15], which directly takes fastq sequences as input. The resulting read counts were used to build an expression matrix, which was then subjected to edgeR analysis. Differentially expressed miRNAs were defined as those miRNA with FDR < 0.05 between two groups of compared samples.

### Analysis of transcriptional changes after incubation of exosomes with cultured HAEC

We generated strand-specific RNA libraries using TruSeq Stranded Total RNA Plus Ribo-zero kits (Illumina). Sequencing was performed at the Genomics and Bioinformatics Core of the State University of New York at Buffalo. Single-end reads per sample were obtained using the HiSeq 2500 platform from Illumina. For mRNA, reads were first trimmed using Cutadap [16] to remove the 3′ end adapters and trailing sequences, followed by aligning to human RefSeq mRNAs (hg19) using TopHat2 [17]. Transcript counts were estimated using HTSeq [18]. Differences in gene expression levels between samples were assessed with edgeR [19].

### Identifying miRNAs associating with individual differentially expressed genes (DEGs)

We first used the miRNA target gene dataset predicted from the algorithm of TargetScanS [20] to find miRNAs that target individual DEGs resulted from RNA profiling analysis. We then searched for the corresponding miRNAs in the miRNA expression dataset, as not all miRNAs from TargetScanS are included in the miRNA expression dataset. The association between individual DEGs and miRNAs from the expression dataset were assessed by plotting their expression changes between JDM and HC.

## Results

### Characterization of exosomes isolated from plasma of children with JDM and controls

**Fig. 1:**
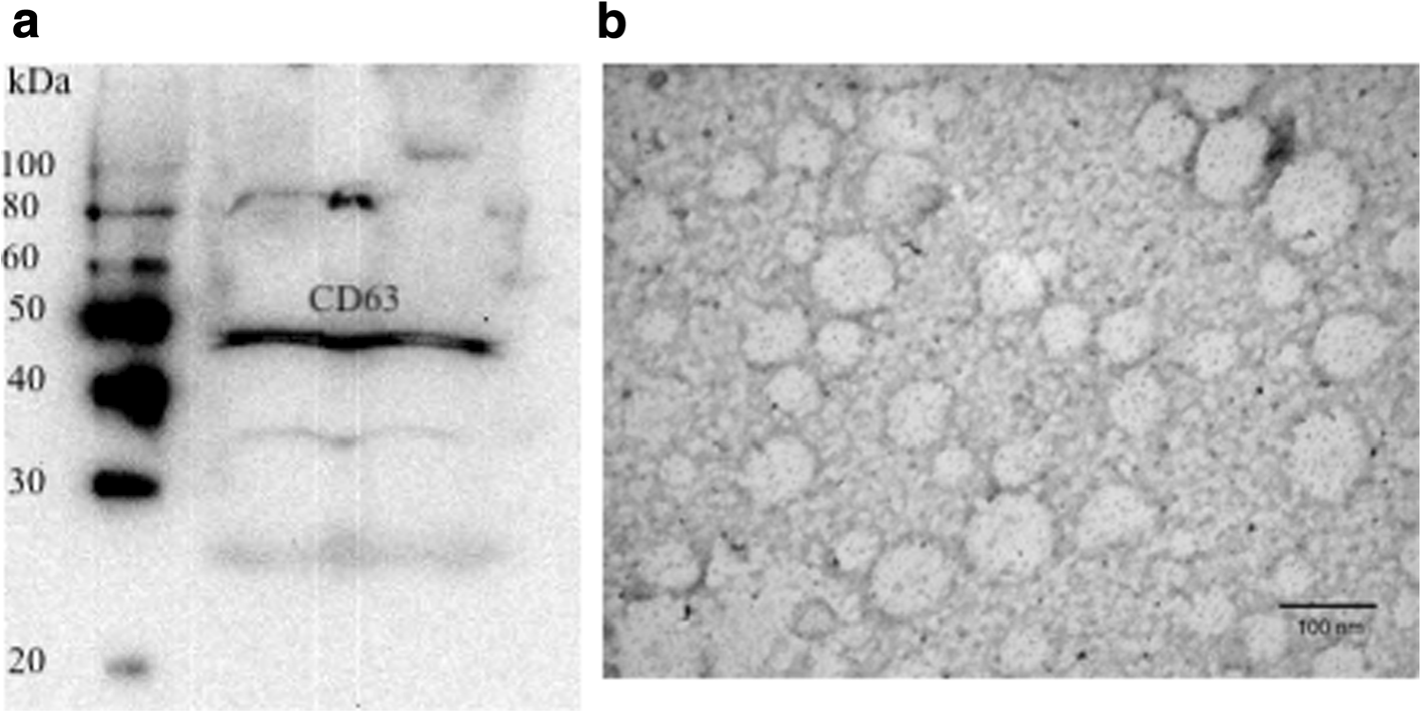
Results of western blotting for CD63 (**a**) and electron micrograph showing size and morphology of exosomes purified from plasma of a child with juvenile dermatomyositis (**b**). Molecular weight markers (100-20 kD) are shown in the first lane of the left panel. The scale for the electron micrograph (100 nm) is shown in the lower right side of the right panel. Exosomes were prepared with the ExoQuick™ reagent from System Biosciences

Figure 1a shows results from the western blotting procedure. All samples were CD63+ by western blotting. Furthermore, exosomes showed the appropriate size and morphology under electron microscopy, as shown in Fig. 1b.

### The small RNA cargoes of JDM-derived exosomes differ from those found in healthy controls

**Fig. 2:**
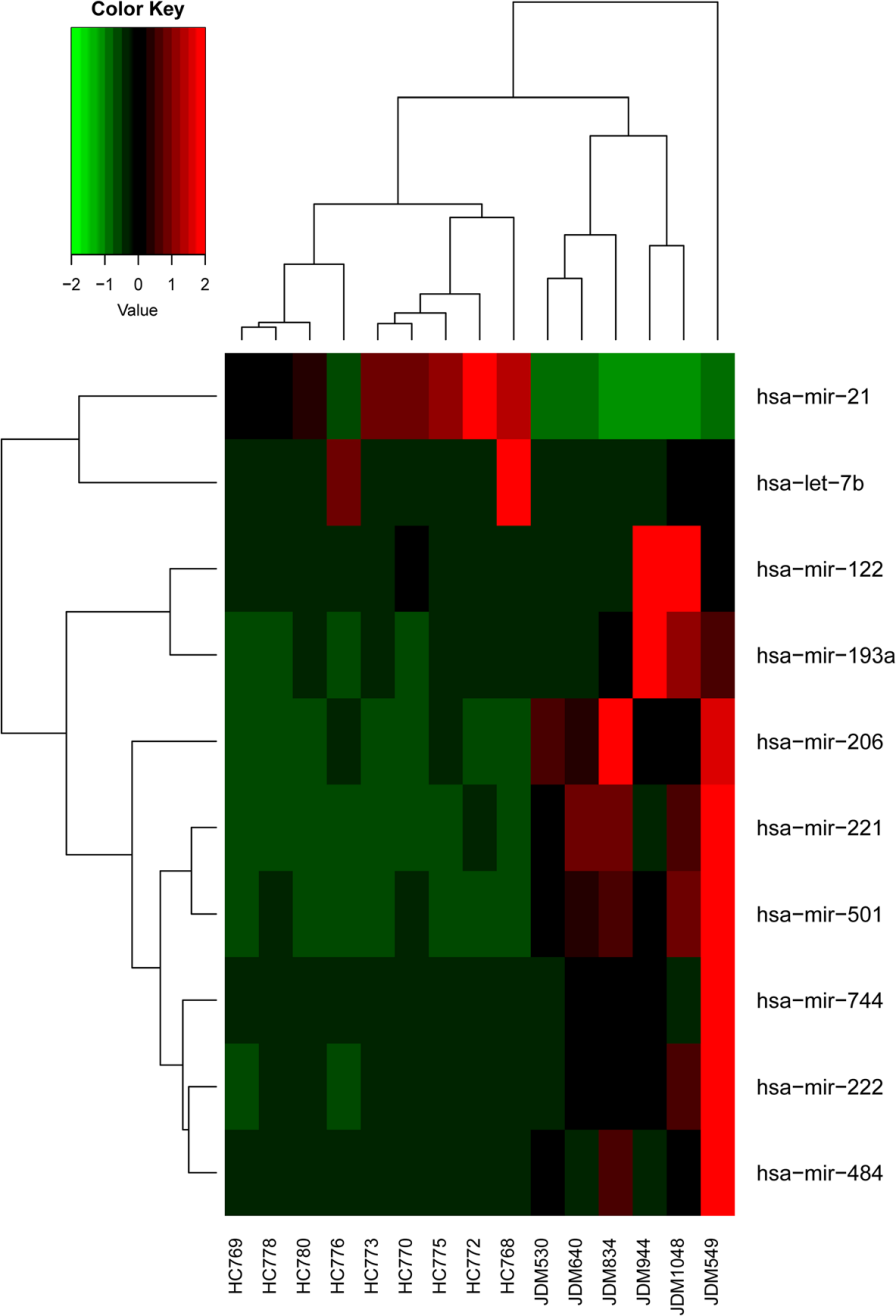
Unsupervised hierarchical clustering analysis of gene expression for the 10 differentially expressed miRNA genes between JDM and HC. The heatmap shows the median-normalized expression of individual miRNA genes across all samples. Heatmap colors represent relative miRNA expression as indicated in the color key

We used small RNA library RNA-seq to compare the differential expression of miRNAs within the exosomes purified from children with JDM and HC. For this analysis, we used 6 JDM and 9 HC exosome samples. Setting the false discovery rate (FDR) at < 0.05, we identified 10 differentially expressed miRNAs in the comparison between JDM and HC, of which 2 show down-regulation, and 8 show up-regulation (Table 1). Hierarchical cluster analysis classified the JDM and HC samples into distinct clusters (Fig. 2).

**Table 1:** Differentially expressed miRNAs between JDM and HC

miRNA	logFC	Fold change	*p* value	FDR
hsa-mir-21	−6.34	−80.84	5.28E-27	7.18E-25
hsa-mir-221	2.55	5.86	4.59E-09	3.12E-07
hsa-mir-206	3.77	13.64	3.90E-04	1.77E-02
hsa-mir-122	2.47	5.56	6.85E-04	1.96E-02
hsa-mir-222	1.37	2.59	7.21E-04	1.96E-02
hsa-let-7b	−3.53	−11.56	9.86E-04	2.23E-02
hsa-mir-501	2.37	5.15	1.32E-03	2.51E-02
hsa-mir-744	2.60	6.05	1.48E-03	2.51E-02
hsa-mir-193a	1.45	2.74	2.64E-03	3.99E-02
hsa-mir-484	1.21	2.32	3.57E-03	4.85E-02

The RNA cargoes that we identified as showing higher abundance in JDM exosome samples (compared to controls) showed no overlap with small RNAs that show higher abundance in plasma exosomes of children with untreated polyarticular JIA. We show results from 40 children with untreated polyarticular JIA and 20 healthy controls in Additional file 1: Table S1. This work is part of an ongoing investigation and will be published in more detail separately.

### AEC take up JDM exosomes; transcriptional profiles differ between cells incubated with JDM exosomes and exosomes from healthy children

**Fig. 3:**
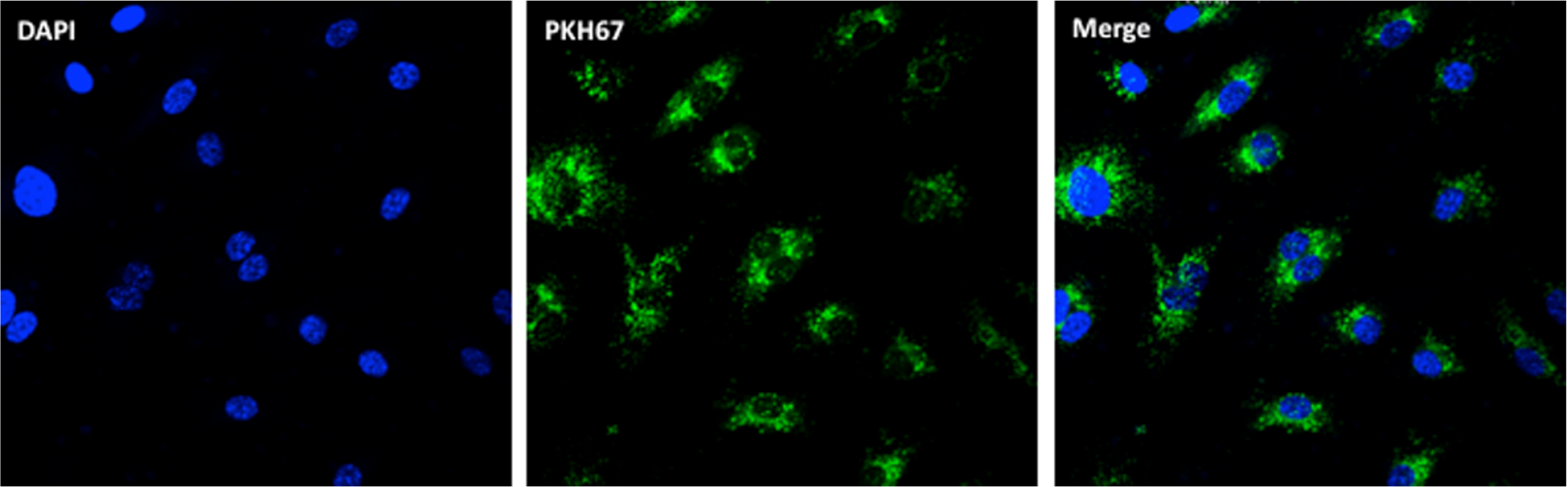
Uptake of exosomes by cultured human aortic endothelial cells (HAEC). HAECs were incubated with PKH67 labeled exosomes (Green) for 24 h. HAECs were fixed by 4% formaldehyde and the slide was mounted using ProLong Gold antifade reagent with DAPI (Blue). The chamber slides and exosomes were visualized with a fluorescent imaging microscope

We next sought to determine whether plasma exosomes might be taken up by HAEC, and whether we could identify transcriptional alterations within HAEC that could be attributed to the RNA cargoes within the JDM exosomes. Figure 3 shows the results of the uptake experiments. Exosomes isolated from both JDM and healthy control plasma were seen within the cytoplasm of HAEC within 24 h hours of exposure.

**Fig. 4:**
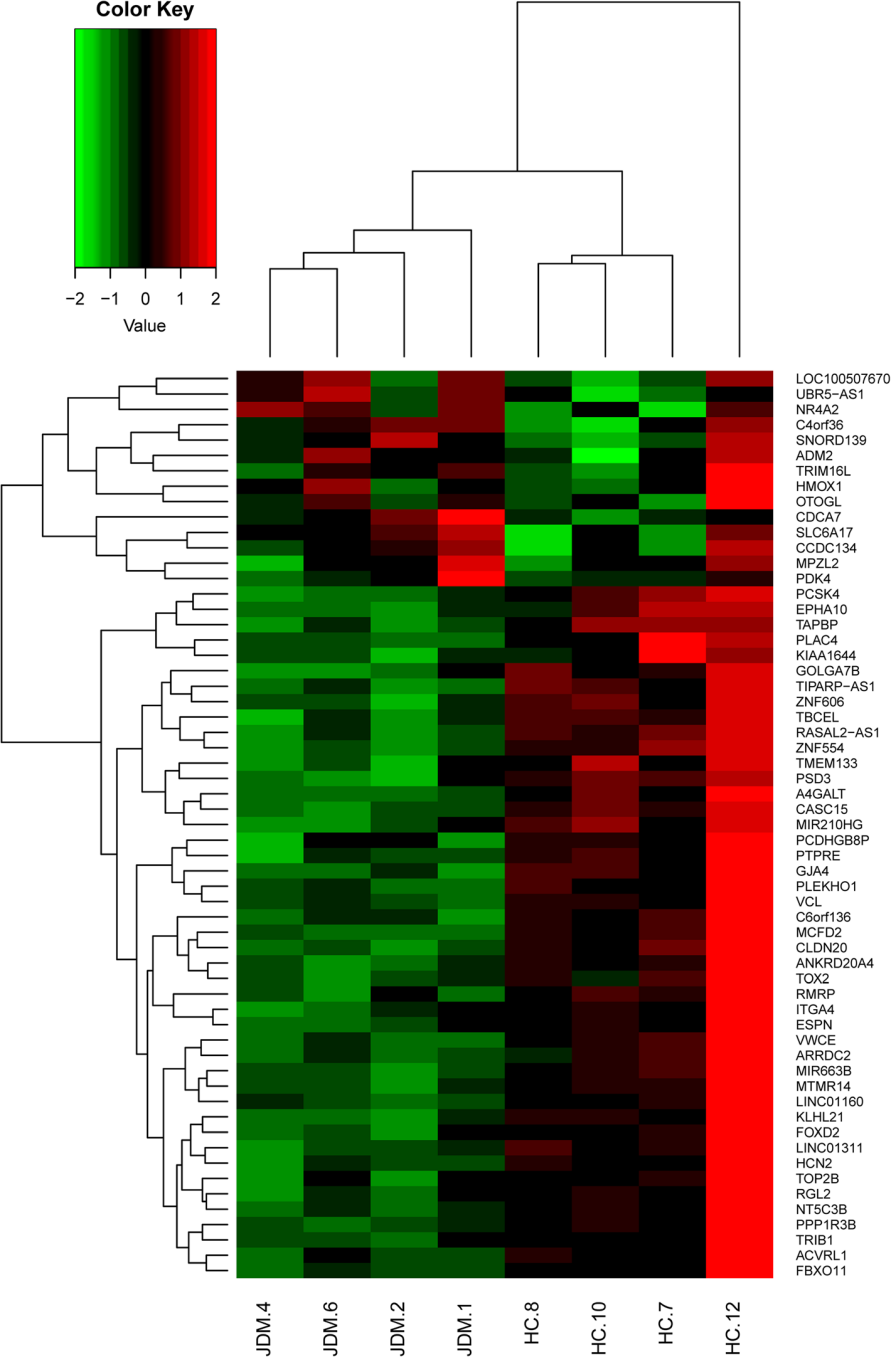
Unsupervised hierarchical clustering analysis of gene expression for the 59 differentially expressed genes between JDM and HC. The heatmap shows the median-normalized expression of individual genes across all samples. Heatmap colors represent relative mRNA expression as indicated in the color key

We next used RNA-seq to compare the expression of genes in HAEC incubated with exosomes from children with untreated JDM (*n* = 4) JDM and HAEC incubated with exosomes from HC (*n* = 4). Setting the cutoff off *p* < 0.01 and fold change > 1.3, we identified 59 differentially expressed genes (DEGs) when we compared HAEC incubated with JDM exosomes with HAEC incubated with exosomes from HC. Of these 59 genes, 45 show higher expression in HAEC incubated with HC exosomes, and 14 show lower expression when compared to HAEC incubated with JDM exosomes (Table 2). Hierarchical cluster analysis using the expression data of the 59 genes from both JDM and HC was able to classify the HAEC incubated with exosomes from JDM samples into a distinct cluster from those incubated with HC exosomes. The later formed one major cluster with three of the 4 samples, as shown in the heatmap (Fig. 4).

**Table 2:** Differentially expressed genes after incubation with HAEC

Gene smybol	Fold change	*p* value	miRNA target^a^
TBCEL	−2.225083292	0.000232193	Yes
TOX2	−2.043385398	0.001375425	Yes
PCDHGB8P	−2.019271064	0.008283468	no
EPHA10	− 1.937909029	0.003578788	Yes
TMEM133	−1.913578811	0.006586193	Yes
A4GALT	− 1.893256114	0.003843604	no
ZNF606	−1.892954092	0.003339793	Yes
C6orf136	−1.884378467	0.002680209	Yes
MIR663B	−1.880005391	0.006120389	no
CLDN20	−1.854101267	0.006351915	no
PTPRE	−1.853862355	0.006160045	Yes
TAPBP	−1.764985627	0.006569879	Yes
TOP2B	−1.731232787	0.001904384	Yes
PCSK4	−1.731028601	0.000191937	no
KIAA1644	−1.713535532	0.006150785	Yes
ARRDC2	−1.708274585	0.005457507	Yes
RMRP	−1.599489386	0.003469351	Yes
PLAC4	−1.590352236	0.001600752	no
MTMR14	−1.566438827	0.004540765	Yes
RGL2	−1.562608346	0.003102877	Yes
TIPARP-AS1	−1.532284267	0.000747686	no
PLEKHO1	−1.527310688	0.00386381	Yes
ITGA4	−1.502769183	0.002712255	Yes
ESPN	−1.489929407	0.000536184	Yes
PPP1R3B	−1.484443928	0.00593945	Yes
MCFD2	−1.463147761	0.009630371	Yes
GOLGA7B	−1.460005241	0.009801722	Yes
VCL	−1.442300805	0.001415881	Yes
NT5C3B	−1.436375137	0.008060946	Yes
ANKRD20A4	−1.431959897	0.006739324	no
ACVRL1	−1.424058578	0.003299645	Yes
GJA4	−1.413440473	0.005108807	no
FBXO11	−1.411318412	0.003194483	Yes
CASC15	−1.407311311	0.000150042	no
FOXD2	−1.396058124	0.001731055	Yes
LINC01311	−1.387030428	0.003652969	no
KLHL21	−1.376156001	0.001415717	Yes
PSD3	−1.365960862	0.003093348	Yes
RASAL2-AS1	−1.34551208	0.00966025	no
TRIB1	−1.336306596	0.008051487	Yes
LINC01160	−1.319909279	0.0099622	no
VWCE	−1.31119374	0.005042249	no
ZNF554	−1.310679787	0.002771376	no
HCN2	−1.300708485	0.00084889	Yes
MIR210HG	−1.30045409	0.006892233	no
TRIM16L	1.302986687	0.000129035	no
HMOX1	1.309164138	0.00026656	Yes
OTOGL	1.314890882	0.003869475	Yes
MPZL2	1.316672807	8.40E-06	Yes
ADM2	1.436182568	0.003847478	Yes
CCDC134	1.465832907	0.001426697	Yes
PDK4	1.545392888	0.002047088	Yes
SNORD139	1.584940011	0.002580409	no
LOC100507670	1.670751157	0.004775702	no
UBR5-AS1	1.744471642	0.004954936	no
SLC6A17	1.751169685	0.008800857	Yes
NR4A2	1.969010915	0.001416847	Yes
CDCA7	2.099733467	0.003238882	Yes
C4orf36	2.128247182	0.00275716	Yes

^a^based on the prediction of Target Scan

We next asked if the genes that showed differential expression in HAEC after incubation with exosomes could be grouped by identifiable biological functions. We used gene ontology features of Biological Process Ontology from GSEA (http://software.broadinstitute.org/gsea/msigdb/index.jsp) to identify specific biological pathways that are altered in HAEC incubated with JDM exosomes. The results indicated that, although no biological functions were linked to the DEGs that showed higher expression in HAEC incubated with JDM exosomes, DEGs with that showed lower expression were associated with a broad range of physiologic functions. Those include cell migration, intercellular junction assembly, cytoplasm organization and biogenesis, and cell-cell adhesion (Additional file 2: Table S2).

### Correlation between miRNA cargoes and patterns of gene expression in HAEC

We next looked for evidence that the miRNAs cargoes of JDM exosomes have direct impact on gene expression within HAEC cells. We first undertook an analysis to determine whether the DEGs identified in the HAEC RNAseq experiments were enriched for target genes of the differentially expressed miRNAs. No significant enrichment was observed, indicating that individual miRNAs have no significant impact on the expression of these DEGs.

However, individual miRNAs can mildly down-regulate hundreds of targets by direct or indirect effects, providing a mechanism of fine-tuning for endothelial gene expression. Furthermore, individual genes can be the targets for multiple miRNAs. Our previous studies have shown that miRNA binding sites have an additive effect on mRNA stability [21]. In our previous studies we demonstrated that the number of miRNAs binding to specific transcripts influences the mRNA decay rate in those miRNA target genes. We found mRNA degradation was enhanced through an additive effect from multiple miRNA targeting [17].

**Fig. 5:**
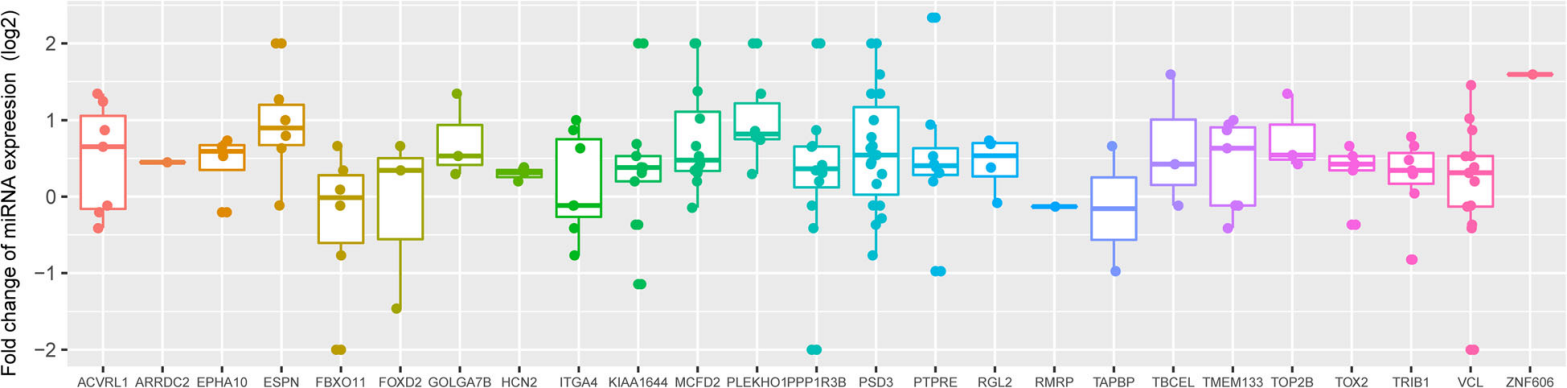
Distribution of fold changes between JDM and HC for miRNAs (each dot represents one miRNA) targeting the corresponding DEGs, which are listed on the x axis. Fold change above zero stands for up-regulation of miRNAs in JDM, when compared to their expression in HC

We therefore undertook a second analysis to determine whether the pattern of DEG expression identified in HAEC incubated with JDM vs HC exosomes might reflect gene regulation by multiple miRNAs instead of the effects of individual miRNAs on individual genes. As shown in Fig. 5, for genes that showed lower expression in HAEC incubated with exosomes from children with JDM (compared to exosomes from HC), this is indeed the case. The majority (22 out of 25) of the DEGs have corresponding up-regulated miRNA abundance in JDM exosomes compared to exosomes from HC.

We did not observe negative association between up-regulated DEGs and corresponding miRNAs (data not shown). Since the majority of the DEGs exhibit lower expression and control a broader range of physiologic functions, the findings indicate that miRNAs from the exosomes of children with with JDM, when taken up by HAEC, impact gene expression within those cells in an additive manner.

## Discussion

Vascular and perivascular inflammation of the skin and muscles are the most prominent features of the pathology of JDM and are like to be directly related to the tissue injury and dysfunction that characterize the clinical disease [22]. Indeed, one of the first comprehensive English language descriptions of the clinical features and natural history of the entity we call “juvenile dermatomyositis” referred to the illness as “systemic angiopathy” of childhood. How and why the vasculature of the skin and muscle (and other organs) become targets of the brisk immune/inflammatory response remains one of the most important unanswered questions with regard to this disease.

Exosomes are one of several families of microparticles that are released from cells either after specific stimuli or during cellular apoptosis. We are now coming to understand that these subcellular components contain small, non-coding RNA molecules that form a previously unrecognized level of transcriptional control in mammals and possibly in simpler organisms [9]. The RNA molecules contained within exosomes and other microparticles are, among other things, a mechanism through which cells of the immune system communicate with one-another [10]. These microparticles are abundant in serum and plasma and have been a source of considerable interest as biomarkers in a broad range of diseases [11–13].

In this paper, we show that exosomes purified from the plasma of children with untreated JDM carry different small RNA cargoes compared to those purified from healthy control children’s plasma. These exosomes can be taken up by HAEC and are associated with altered gene expression in those cells. Furthermore, endothelial cell genes whose expression is altered by these exosomes regulate basic and essential functions for endothelial cells, including include cell migration, intercellular junction assembly, cell-cell adhesion, and cytoplasm organization and biogenesis. Furthermore, computational analysis that incorporates the additive effects of multiple miRNA on gene expression [17] support the idea that the transcriptional effects are due to the RNA cargoes within the JDM exosomes.

There are several limitations to this study that need to be considered in interpreting the data. The first is the small number of samples studied. While there was considerable homogeneity in the RNA cargoes found in the plasma exosomes of healthy children (Fig. 2), there was considerable inter-subject variability within the JDM exosome samples. This finding is consistent with the broad range of clinical phenotypes subsumed within the single disease entity, JDM. This limitation prevents us from making broad generalizations regarding the full range of miRNA cargoes within JDM exosomes or associating specific miRNA with specific clinical phenotypes. However, we believe that despite the small numbers, these findings are sufficient to establish that there are real differences in the miRNA cargoes of exosomes derived from children with with JDM. Furthermore, the biological effects of these exosomes, as reflected in the transcriptomes of HAEC after they have taken up the exosomes, are quite uniform (Fig. 4) and demonstrate cogent biological effects. We believe that further efforts to categorize the small RNA content of JDM exosomes and to relate specific small RNAs to particular clinical features could provide useful clinical and pathological insights.

A second limitation of the study is the cells that we used. We chose AEC because we had used them in our earlier work examining anti-endothelial cell antibodies (AECA) in JDM [7]. However, JDM pathology is most prominent in small arterial vessels [22], although larger vessels can be involved. It is possible that the patterns of gene expression would have differed if we had used a different cell line.

Despite these limitations, we believe that these simple experiments provide the basis for further inquiries into the mechanisms leading to the targeting of blood vessels by immune/inflammatory cells in JDM. Understanding this process better can be expected to provide the foundation for new therapeutic strategies.

## Conclusions

The RNA cargoes carried by plasma exosomes of children with JDM differ from those found in healthy children. These exosomes are taken up by endothelial cells and influence the transcriptional program in these cells. These findings suggest a previously unsuspected mechanism underlying the targeting of the vasculature in JDM.

## Additional files

Additional file 1: **Table S1.** Small RNAs Showing Differential Abundance in Plasma Exosomes of Children With Polyarticular JIA. (DOCX 20 kb)

Additional file 2: **Table S2.** Ontology analysis for down- and up regulated DEGs. (DOCX 18 kb)

## Data Availability

We are now preparing both the mRNAseq and small RNAseq data for public availability on the Gene Expression Omnibus (GEO) website.

## Abbreviations

DEGs: Differentially expressed genes
HAEC: Human aortic endothelial cells
HC: Healthy controls
JDM: Juvenile dermatomyositis
RNAseq: RNA sequencing
