# Supplementary material for: Plasma exosomes from children with juvenile dermatomyositis are taken up by human aortic endothelial cells and are associated with altered gene expression in those cells
Source: Pediatr Rheumatol Online J. 2019 Jul 12;17:41. doi: 10.1186/s12969-019-0347-0 (PMC6626431; doi:10.1186/s12969-019-0347-0)
Supplement: Supplementary file 1 — Table S1. Small RNAs Showing Differential Abundance in Plasma Exosomes of Children With Polyarticular JIA. (DOCX 20 kb) [file 12969_2019_347_MOESM1_ESM.docx]

**Additional file 1: Table S1: Small RNAs Showing Differential Abundance in Plasma Exosomes of Children**

**With Polyarticular JIA**

| ***ID*** | ***logFC*** | ***logCPM*** | ***PValue*** | ***FDR*** |
| --- | --- | --- | --- | --- |
| snR39B | -1.45248528 | 7.560807506 | 0.000104912 | 0.024911865 |
| hsa-mir-328 | 1.431334604 | 8.326252709 | 0.000180697 | 0.024911865 |
| hsa-mir-1260b | 1.709467965 | 7.700628331 | 0.000222865 | 0.024911865 |
| U105 | -1.855009935 | 6.572969831 | 0.000247816 | 0.024911865 |
| U59A | -1.856872343 | 6.929688475 | 0.000295865 | 0.024911865 |
| hsa-mir-423 | 1.016280612 | 11.83597847 | 0.000519457 | 0.034215717 |
| hsa-mir-326 | 1.976264512 | 7.423909521 | 0.000568907 | 0.034215717 |
| hsa-mir-409 | 2.36133234 | 7.697681298 | 0.00086324 | 0.041250992 |
| hsa-mir-1273 | 1.232730002 | 9.170364552 | 0.00088185 | 0.041250992 |
| 14q(II-3) | -1.216119261 | 8.278817015 | 0.001066806 | 0.044912537 |
| hsa-mir-323b | 3.397854845 | 7.041194744 | 0.00126122 | 0.048270332 |
| hsa-mir-886 | -1.158335012 | 8.205257295 | 0.001478186 | 0.051859675 |
| U75 | -1.892559427 | 6.591282455 | 0.002178041 | 0.070535022 |
| hsa-mir-146a | 1.08261866 | 10.215437 | 0.003187085 | 0.084656507 |
| hsa-mir-130a | 1.28807516 | 9.440274305 | 0.003351489 | 0.084656507 |
| U27 | 1.076470001 | 9.837861268 | 0.003357273 | 0.084656507 |
| hsa-mir-564 | 0.958447903 | 8.215011784 | 0.003418434 | 0.084656507 |
| hsa-mir-137 | -1.277438449 | 6.99413059 | 0.006469249 | 0.151308541 |
| HBII-135 | -1.738330086 | 7.252165206 | 0.007918307 | 0.175453023 |
