# Supplementary material for: Plasma exosomes from children with juvenile dermatomyositis are taken up by human aortic endothelial cells and are associated with altered gene expression in those cells
Source: Pediatr Rheumatol Online J. 2019 Jul 12;17:41. doi: 10.1186/s12969-019-0347-0 (PMC6626431; doi:10.1186/s12969-019-0347-0)
Supplement: Supplementary file 2 — Table S2. Ontology analysis for down- and up regulated DEGs. (DOCX 18 kb) [file 12969_2019_347_MOESM2_ESM.docx]

| **Additional file 2: Table S2: Ontology analysis for down- and up regulated DEGs** | |  | |  | |
| --- | --- | --- | --- | --- | --- |
| **Enriched biological process** | **p-values (down-regulate genes)** | | **p-values (up-regulated genes)** | |  |
| NEGATIVE_REGULATION_OF_CELL_MIGRATION | 6.34E-04 | | na | |  |
| INTERCELLULAR_JUNCTION_ASSEMBLY | 1.01E-03 | | na | |  |
| INTERCELLULAR_JUNCTION_ASSEMBLY_AND_MAINTENANCE | 1.23E-03 | | na | |  |
| CYTOPLASM_ORGANIZATION_AND_BIOGENESIS | 1.48E-03 | | na | |  |
| REGULATION_OF_CELL_MIGRATION | 2.02E-03 | | na | |  |
| PROTEIN_COMPLEX_ASSEMBLY | 8.02E-03 | | na | |  |
| CELL_CELL_ADHESION | 1.04E-02 | | na | |  |
| CELL_MIGRATION | 1.45E-02 | | na | |  |
| PROTEIN_METABOLIC_PROCESS | 2.95E-02 | | na | |  |
| MACROMOLECULAR_COMPLEX_ASSEMBLY | 4.30E-02 | | na | |  |
| CELLULAR_COMPONENT_ASSEMBLY | 4.84E-02 | | na | |  |
